# Роль кальций-чувствительного и витамин D рецепторов в патогенезе множественного поражения околощитовидных желез при спорадическом первичном гиперпаратиреозе

**DOI:** 10.14341/probl13207

**Published:** 2023-06-30

**Authors:** Е. A. Ильичева, И. А. Шурыгина, Н. Н. Дремина, Г. А. Берсенев, Е. Г. Григорьев

**Affiliations:** Иркутский научный центр хирургии и травматологии; Иркутский научный центр хирургии и травматологии; Иркутский научный центр хирургии и травматологии; Иркутский научный центр хирургии и травматологии; Иркутский научный центр хирургии и травматологии; Иркутский государственный медицинский университет

**Keywords:** множественное поражение околощитовидных желез, солитарная аденома, первичный гиперпаратиреоз, патогенез, кальций-чувствительный рецептор, рецептор витамина D

## Abstract

**ОБОСНОВАНИЕ:**

ОБОСНОВАНИЕ. Множественное поражение околощитовидных желез (ОЩЖ) составляет ¼ наблюдений первичного гиперпаратиреоза (ПГПТ). Однако унифицированные диагностический и лечебный алгоритмы при данном варианте ПГПТ до сих пор не разработаны. Одно из возможных направлений поиска решений этой проблемы — исследование молекулярно-генетических особенностей заболевания и ассоциированных с ними клинико-лабораторных факторов.

**ЦЕЛЬ:**

ЦЕЛЬ. Изучить особенности экспрессии кальций-чувствительного (CaSR) и витамин D (VDR) рецепторов на поверхности паратиреоцитов при ПГПТ с солитарным и множественным поражением ОЩЖ, а также ее динамики при снижении фильтрационной функции почек.

**МАТЕРИАЛЫ И МЕТОДЫ:**

МАТЕРИАЛЫ И МЕТОДЫ. В одноцентровое наблюдательное проспективное исследование с ретроспективным сбором данных включены пациенты, оперированные по поводу ПГПТ, вторичного (ВГПТ) в 2019–2021 гг. и третичного гиперпаратиреоза (ТГПТ) в 2014–2021 гг. Изучалась экспрессия CaSR, VDR и ее взаимосвязь с основными лабораторными показателями, клиническим вариантом гиперпаратиреоза, морфологическим субстратом.

**РЕЗУЛЬТАТЫ:**

РЕЗУЛЬТАТЫ. В исследование включены 19 пациентов с множественным и 25 с солитарным поражением ОЩЖ при ПГПТ; 15 с ВГПТ и 10 с ТГПТ (69 человек). Статистически значимое снижение частоты выявления нормальной экспрессии рецепторов CaSR и VDR встречается при любом морфологическом варианте гиперпаратиреоза и отмечается в 93–60% препаратов. Снижение нормальной экспрессии CaSR при гиперплазии выявляется значимо реже, чем при аденоме (р≤0,01). Медиана интенсивности экспрессии при аденоме составила 2,5 (2–3), при гиперплазии — 3,5 (3–4) (р≤0,01). Различие молекулярно-генетических механизмов развития гиперпаратиреоза при солитарной аденоме и множественном поражении ОЩЖ проявляется в частоте сохранения нормальной экспрессии CaSR в ткани ОЩЖ. Перечисленные механизмы реализуются на локальном уровне, их вариабельность не изменяется под влиянием заместительной почечной терапии (ЗПТ). Общей молекулярно-генетической закономерностью развития гиперпаратиреоза с преобладанием морфологического субстрата (аденома или гиперплазия) следует считать снижение частоты сохранения нормальной экспрессии VDR в ОЩЖ (до 7–13%) (p<0,01). Этот механизм реализуется на локальном уровне, его вариабельность изменяется под влиянием ЗПТ, достигая статистически значимых различий у больных ТГПТ.

**ЗАКЛЮЧЕНИЕ:**

ЗАКЛЮЧЕНИЕ. Исследование демонстрирует особенности изменения экспрессии CaSR и VDR при ПГПТ с множественным поражением ОЩЖ. Показана взаимосвязь экспрессии этих рецепторов с клиническим вариантом гиперпаратиреоза, морфологическим субстратом, основными лабораторными показателями и почечной функцией.

## Abstract (en)

**BACKGROUND:**

BACKGROUND: Sporadic multiple parathyroid gland disease is ¼ cases of primary hyperparathyroidism (PHPT). However, a single tactic for diagnosing and operating volume in patients with this variant of PHPT has not yet been developed. One of the possible directions in the search for pathogenetically substantiated methods of diagnosis and treatment is the study of the molecular genetic features of the disease and associated clinical and laboratory factors.

**AIM:**

AIM: To study the features of the expression of calcium sensitive (CaSR) and vitamin D (VDR) receptors on the surface of parathyroid cells in primary hyperparathyroidism with solitary and multiple lesions of the parathyroid glands, as well as its changes under the influence of a decrease in the filtration function of the kidneys.

**MATERIALS AND METHODS:**

MATERIALS AND METHODS: In a single center observational prospective study with retrospective data collection, there were patients who during 2019–2021. operated on for PHPT, secondary hyperparathyroidism (SHPT) and all cases of tertiary hyperparathyroidism (THPT) operated during 2014–2021. The expression of CaSR, VDR and its relationship with the main laboratory parameters, the clinical variant of hyperparathyroidism, and the morphological substrate were studied.

**RESULTS:**

RESULTS: The study included 69 patients: 19 with multiple and 25 with solitary PTG near PHPT, 15 with SHPT, 10 with THPT. A statistically significant decrease in the frequency of detection of normal expression of CaSR and VDR receptors occurs in any morphological variant of hyperparathyroidism and is observed in 93–60% of drugs. A decrease in the normal expression of CaSR in hyperplasia is detected statistically significantly less frequently than in adenoma (p≤0.01). The median expression intensity in adenoma was 2.5 (2:3), in hyperplasia 3.5 (3–4) (p≤0.01). The difference in the molecular mechanisms of the development of hyperparathyroidism with a predominance of a morphological substrate in the form of adenoma (PHPT with solitary adenoma) or hyperplasia (SHPT and PHPT with multiple PTG lesions) is realized in the frequency of maintaining normal CaSR expression in the PTG tissue. These mechanisms are implemented at the local level, their variability does not change under the influence of RRT. A common molecular genetic mechanism for the development of hyperparathyroidism with a predominance of a morphological substrate in the form of adenoma or hyperplasia has been found to reduce the frequency of maintaining normal VDR expression in PTG (up to 7–13%), p<0.01. This mechanism is implemented at the local level, its variability changes under the influence of RRT, reaching statistically significant differences in patients with THPT.

**CONCLUSION:**

CONCLUSION: The study demonstrates the features of changes in the expression of CaSR and VDR in PHPT with multiple lesions of the parathyroid glands. The relationship between the expression of these receptors and the clinical variant of hyperparathyroidism, the morphological substrate, the main laboratory parameters, and renal function was shown.

## ОБОСНОВАНИЕ

Первичный гиперпаратиреоз (ПГПТ) — распространенное эндокринологическое заболевание, вызванное первичной патологией околощитовидных желез (ОЩЖ) [1].

Преобладает спорадический гиперпаратиреоз (90–95%) (сПГПТ), в 5–10% наблюдений патология встречается в рамках наследственных синдромов. Дебют заболевания до 40 лет подозрителен в отношении наследственного характера заболевания [2]. В 80–85% наблюдений сПГПТ встречается солитарная аденома, в 20–25% — поражение нескольких желез, и менее 1% составляет рак [3]. Специфических признаков множественного поражения ОЩЖ при сПГПТ нет.

Количество оперативных вмешательств по поводу сПГПТ заметно увеличивается, однако унифицированной концепции диагностики и объема хирургического вмешательства при множественном поражении ОЩЖ до сих пор нет. Между тем определение количества избыточно функционирующих ОЩЖ на предоперационном этапе позволяет выбрать оптимальный объем операции, что в главном обеспечивает эффективность лечения сПГПТ.

Одним из направлений поиска патогенетически обоснованных методов диагностики и лечения множественного поражения ОЩЖ при сПГПТ остается исследование молекулярно-генетических особенностей заболевания и ассоциированных с ними клинико-лабораторных факторов.

Нарушение секреции паратиреоидного гормона (ПТГ), контролируемое кальций-чувствительным рецептором (CaSR), и усиленная пролиферация клеток ОЩЖ (паратиреоцитов) имеют ключевое значение для развития сПГПТ [4]. Кроме того, секреторная и пролиферативная активность ОЩЖ через рецептор витамина D (VDR) контролируется кальцитриолом (1,25-дигидроксивитамином D) [5].

Доказано снижение экспрессии CaSR клеток аденомы в 2 раза по сравнению с нормальной тканью ОЩЖ [6]. В эксперименте показано, что CaSR — ключевой фактор, определяющий пролиферацию клеток ОЩЖ, а VDR играет второстепенную роль [7]. Гиперплазия ОЩЖ предшествует подавлению экспрессии CaSR на модели уремических крыс [8], при этом введение кальцитриола или кальцимиметиков приводило к снижению пролиферации клеток. Это связано с повышением экспрессии CaSR и VDR на фоне введенных препаратов [9].

В контексте сПГПТ хронический дефицит витамина D — возможный механизм развития гиперплазии ОЩЖ с последующим приобретением автономной секреции ПТГ и трансформации в аденому [10], ухудшающий течение заболевания. Это связано с высокими уровнями ПТГ и кальция, увеличением массы аденомы, повышением метаболизма костной ткани с более низкой минеральной плотностью костей и риском их малотравматичных переломов [11].

Влияние дефицита витамина D на естественное течение сПГПТ уже не вызывает сомнений, поэтому нельзя отвергать роль уремического фактора.

Длительное время развитие камней в почках и связанные с ними осложнения (инфекция мочевыводящих путей, гидронефроз и почечная недостаточность) считались классическими проявлениями ПГПТ. Однако с введением понятия бессимптомной формы на первый план вышли гиперкальциурия (повышенная экскреция кальция с мочой), снижение фильтрационной и концентрационной функции почек.

По мере снижения фильтрационной функции стимуляция ОЩЖ повышенной концентрацией сывороточного фосфата в сочетании с пониженным содержанием внеклеточного ионизированного кальция и кальцитриола в сыворотке крови приводит к увеличению синтеза ПТГ. Обсуждаемый компенсаторный механизм наблюдается у пациентов со 2-й стадии ХБП, когда скорость клубочковой фильтрации (СКФ) остается в пределах нормы (60–90 мл/мин/1,73 м2). На ранних стадиях развития гиперпаратиреоза эти изменения усиливаются недостаточной экспрессией CaSR и VDR, что делает клетки ОЩЖ неспособными адекватно реагировать на кальций и/или кальцитриол [12].

## ЦЕЛЬ ИССЛЕДОВАНИЯ

Изучить особенности экспрессии кальций-чувствительного и витамин D рецепторов на поверхности паратиреоцитов при ПГПТ с солитарным и множественным поражением ОЩЖ, а также ее изменения при снижении фильтрационной функции почек.

## МАТЕРИАЛЫ И МЕТОДЫ

## Место и время проведения исследования

Клиническое исследование проведено с сентября 2019 г. по март 2021 г. на базе отделения торакальной хирургии ГБУЗ «Иркутская ордена “Знак почета” областная клиническая больница», г. Иркутск.

Морфологическое — с сентября 2019 г. по март 2021 г. на базе отделения общей патологии ГБУЗ «Иркутское областное патологоанатомическое бюро», г. Иркутск.

Иммуногистохимическое — с апреля 2021 г. по январь 2022 г. на базе научно-лабораторного отдела ФГБНУ «Иркутский научный центр хирургии и травматологии» (далее ИНЦХТ), г. Иркутск.

## Методы

В одноцентровое наблюдательное проспективное исследование с ретроспективным сбором данных включены пациенты независимо от пола и возраста, которые в течение 2019–2021 гг. прооперированы по поводу сПГПТ, вторичного гиперпаратиреоза (ВГПТ), а также третичного гиперпаратиреоза (ТГПТ) в течение 2014–2021 гг.

Критерий включения — показания к хирургическому лечению сПГПТ в соответствии с клиническими рекомендациями [13], а также к хирургическому лечению ВГПТ у пациентов, находящихся на заместительной почечной терапии гемодиализом (ЗПТ ГД), в соответствии с рекомендациями Российской ассоциации эндокринологов 2006, 2013 гг. и Национальными рекомендациями по минеральным и костным нарушениям при хронической болезни почек Российского диализного общества 2011 г. [14] и к хирургическому лечению ТГПТ на заместительной почечной терапии трансплантацией почки (ЗПТ ТП) [15][16]. Критерий исключения — возраст моложе 40 лет в сочетании с ПГПТ.

Обследование включало демографические показатели (пол, возраст), биохимические показатели крови (креатинин с расчетом СКФ по формуле CKD-EPI (2011), общий и ионизированный кальций, альбумин), уровень паратиреоидного гормона и витамина D (кальцидиола), суточную экскрецию кальция и фосфора с мочой. Выполнялось ультразвуковое исследование (УЗИ) органов брюшной полости, а также остеоденситометрия с определением T-критерия для верификации формы ПГПТ. Анатомические особенности ОЩЖ оценивали на основании УЗИ и сцинтиграфии с использованием радиофармпрепарата 99mTc-технетрил в сочетании с однофотонной эмиссионной компьютерной томографией. При подозрении на множественное поражение ОЩЖ проводилась мультиспиральная компьютерная томография шеи с внутривенным контрастным усилением. Для определения выраженности костных нарушений с количественной оценкой минеральной плотности кости выполняли остеосцинтиграфию с 99mTc-пирфотехом.

Морфологический материал ткани ОЩЖ получен в ходе оперативных вмешательств пациентам по поводу ПГПТ, ВГПТ на ЗПТ ГД и ТГПТ на ЗПТ ТП. В исследование включены результаты световой микроскопии и иммуногистохимической окраски 140 препаратов ткани ОЩЖ на CaSR и VDR. Поскольку известно, что наибольшая экспрессия обоих рецепторов наблюдается в нормальных тканях ОЩЖ, то для ранжирования экспрессии была выполнена интраоперационная биопсия 1/3 части унилатеральной визуально неизмененной железы у 5 пациентов с ПГПТ при удалении солитарной аденомы. Нормальность строения этих желез была подтверждена в гистологическом исследовании.

Морфологическое исследование включало рутинную световую микроскопию окрашенных гематоксилином-эозином удаленных ОЩЖ.

Для проведения иммуногистохимического исследования использовали парафиновые срезы ОЩЖ. Применялся непрямой метод, который основан на двухэтапном нанесении специфичных и антивидовых антител. В качестве первичных использовались кроличьи антитела к CaSR и VDR:

В качестве вторичных — меченные пероксидазой хрена козьи антитела к кроличьим иммуноглобулинам: NovolinkPolymer, Anti-rabbit Poly-HRP-IgG (RE 7112 из набора Novolink Polymer Detection System RE7140-K). Иммуногистохимическое исследование выполняли в соответствии с протоколом, предлагаемым производителем первичных антител компанией Abcam1.

Микроскопическое исследование проводили с использованием светового микроскопа CarlZeiss Axio Vert. A1 и регистрировали на камеру AxioCam ICc5 с программным обеспечением CarlZeiss Zen 2.3.

Предметные стекла исследовали под световым микроскопом при увеличении ×20 в 10 полях зрения. Проводился качественный анализ экспрессии CaSR и VDR с распределением результатов на 4 степени интенсивности окраски (рис. 1): 3-я — высокая экспрессия рецептора; 2-я — умеренная экспрессия рецептора; 1-я — низкая экспрессия рецептора; 0-я — отсутствие экспрессии рецептора. Анализ проводился тремя независимыми исследователями с подсчетом среднего результата.

Дизайн морфологического и иммуногистохимического исследования представлен в таблице 1.

**Figure:**
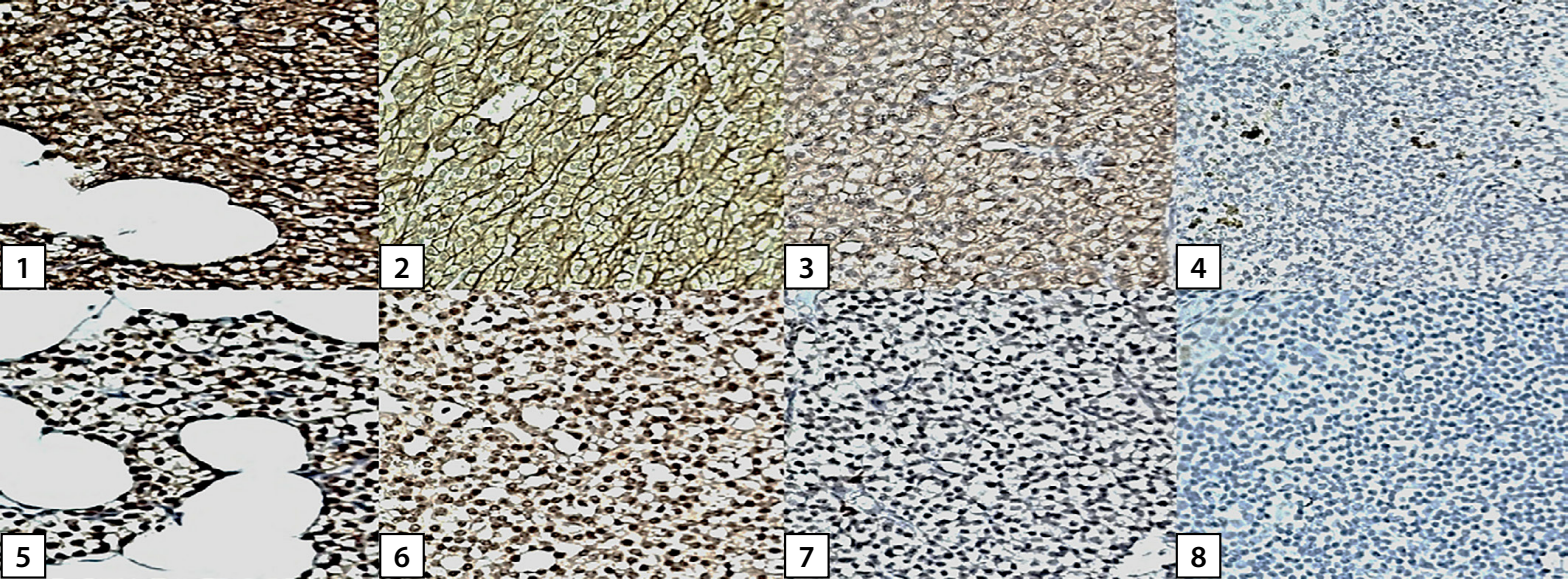
Рисунок 1. Качественный анализ экспрессии CaSR и VDR, основанный на интенсивности окраски.Примечание. CaSR (1–4) экспрессируется в основном на межклеточной границе паратиреоцитов, а VDR (5–8) — в основном в ядре. Степени окраски: 3-я степень — 1 и 5; 2-я степень — 2 и 6; 1-я степень — 3 и 7; 0-я степень — 4 и 8.Figure 1. Qualitative analysis of CaSR and VDR expression based on stain intensity

**Table:** Таблица 1. Дизайн морфологического и иммуногистохимического исследованияTable 1. Design of morphological and immunohistochemical study Примечание. Категориальные данные представлены в виде числа наблюдений и частоты в процентах, n (%). * — в основной группе у 8 пациентов на иммуногистохимическое исследование исследование взят 1 парафиновый блок, поскольку множественное поражение ОЩЖ установлено в послеоперационном периоде.

Группы исследования	Количество парафиновых блоков от одного пациента	Количество препаратов, окрашенных на CaSR и VDR nпрепаратов =140 (100%)
количество блоков	количество пациентов
Основная группа — множественное поражение ОЩЖ при сПГПТ nпациентов =19 (100%)	1*	8 (42,1)*	8 (5,9)*
2	10 (52,6)	20 (14,8)
3	1 (5,3)	3 (2,2)
Группа сравнения 1.1 — солитарное поражение ОЩЖ при сПГПТ с уровнем СКФ>60 мл/мин/1,73 м2 nпациентов=16 (100%)	1	16 (100)	16 (11,8)
Группа сравнения 1.2 — солитарное поражение ОЩЖ при сПГПТ с уровнем СКФ<60 мл/мин/1,73 м2 nпациентов=9 (100%)	1	9 (100)	9 (6,6)
Группа сравнения 2 — ВГПТ на ЗПТ ГД nпациентов=15 (100%)	2	1 (6,8)	2 (1,65)
3	10 (66,6)	30 (22,2)
4	4 (26,6)	16 (11,8)
Группа сравнения 3 — ТГПТ на ЗПТ ТП nпациентов=10 (100%)	2	1 (10)	2 (1,65)
3	7 (70)	21 (15,5)
4	2 (20)	8 (5,9)
Неизмененная ткань ОЩЖ, взятая в результате интраоперационной биопсии интактной ОЩЖ у пациентов с солитарным поражением ОЩЖ и СКФ>60 мл/мин/1,73 м2 nпациентов=5 (100%)	1	5 (100)	5 (3,7)

## Статистический анализ

Статистический анализ проводили с помощью пакета программ Statistica 10.0 for Windows (лицензия №AXAR402G263414FA-V). Выполнялся описательный и сравнительный анализ с использованием методов непараметрической статистики. Непрерывные данные представляли в виде медианы с нижним и верхним квартилями (IQR — interquartile range, межквартильный диапазон). Определение статистической значимости различий для непрерывных данных (р) в сравниваемых выборках проведено по критериям Манна–Уитни (U), Краскела–Уоллиса, Вилкоксона (W). Определение статистической значимости различий для категориальных данных (р) проведено по критериям Пирсона (χ2), точного критерия Фишера. Корреляционный анализ выявленных статистически значимых параметров проведен на основе ранговых корреляций Спирмена. Многофакторный анализ проведен методом множественной нелинейной регрессии.

## Этическая экспертиза

Все пациенты подписали информированное согласие на участие в исследовании. Исследование одобрено комитетом по биомедицинской этике ИНЦХТ, протокол №8 от 23.12.2019 г.

## РЕЗУЛЬТАТЫ

В соответствии с критериями в исследование случайным образом включены 69 пациентов. Основная группа — множественное поражение ОЩЖ при ПГПТ (n=19); сравнения: 1 — солитарное поражение ОЩЖ при ПГПТ (1.1 — при СКФ>60 мл/мин/1,73 м2 (n=16); 1.2 — при СКФ<60 мл/мин/1,73 м2 (n=9)), 2 — множественное поражение ОЩЖ при ВГПТ на ЗПТ ГД (n=15), 3 — множественное поражение ОЩЖ при ТГПТ на ЗПТ ТП (n=10). В таблице 2 представлены клинико-лабораторные показатели, отражающие общую характеристику включенных в исследование пациентов.

Согласно данным таблицы 2, во всех группах преобладали женщины старше 50 лет, кроме ВГПТ на ЗПТ ГД, в которой пациенты моложе и мужчин было больше. У всех больных выявлены нарушения минерального и костного обмена, характерные для соответствующей патологии. В зависимости от группы исследования нарушение фильтрационной функции почки варьировалось от 1-й до 5-й стадии ХБП.

**Table:** Таблица 2. Клинико-лабораторные характеристики основной группы и групп сравненияTable 2. Clinical and laboratory characteristics of the main group and comparison groups Примечание. Непрерывные данные представлены в виде медианы и межквартильного диапазона (IQR). Категориальные данные представлены в виде числа наблюдений и частоты в процентах (абс. (%)). Сокращения: Ca — кальций, Мин — минимальный, ОДМ — остеоденситометрия.

Характеристика	Основнаягруппа Множественное поражение ОЩЖ при ПГПТ n=19 (100%)	Группа сравнения 1.1 Солитарное поражение ОЩЖ при ПГПТ, уровень СКФ>60 мл/мин/1,73 м2 n=16 (100%)	Группа сравнения 1.2 Солитарное поражение ОЩЖ при сПГПТ, уровень СКФ<60 мл/мин/1,73 м2 n=9 (100%)	Группа сравнения 2 ВГПТ на ЗПТ ГД n=15 (100%)	Группа сравнения 3 ТГПТ на ЗПТ ТП n=10 (100%)
Возраст, лет; медиана (IQR)	64,0 (58,0; 67,0)	61,0 (52,5; 63,0)	65,0 (62,0; 68,0)	52,0 (32,0; 59,0)	61,5 (56,0; 65,0)
Число женщин, n (%)	19 (100)	14 (87,5)	9 (100)	4 (26,6)	8 (80)
Ca, скорректированный по альбумину (2,1–2,6 ммоль/л); медиана (IQR)	2,65 (2,50; 2,73)	2,62 (2,51; 2,83)	2,75 (2,70; 2,81)	2,43 (2,26; 2,54)	2,68 (2,58; 2,81)
Ca ионизированный (1,15–1,27 ммоль/л); медиана (IQR)	1,47 (1,34; 1,61)	1,44 (1,41; 1,56)	1,56 (1,51; 1,63)	1,29 (1,23; 1,35)	1,60 (1,49; 2,25)
Кальциурия суточная (2,5–6,25 ммоль/сут); медиана (IQR)	7,32 (3,96; 8,19)	8,16 (5,99; 11,04)	3,53 (3,23; 7,74)	-	1,78 (1,17; 3,56)
ПТГ (15,0–68,3 пг/мл); медиана (IQR)	137,80 (106,70; 260,0)	139,05 (108,80; 248,35)	318,00 (207,10; 489,50)	1828,0 (1223,0; 2340,0)	833,10 (380,0; 1977,55)
Витамин D; медиана (IQR)	23,41 (15,50; 32,13)	21,33 (16,51; 24,47)	17,62 (11,87; 20,49)	24,25 (22,50; 26,70)	22,78 (12,13; 28,00)
Креатинин (менее 0,106 ммоль/л для мужчин, менее 0,08 для женщин); медиана (IQR)	84,00 (80,00; 96,00)	74,50 (64,50; 84,50)	94,00 (90,0; 107,0)	925,00 (770,0; 1110,0)	195,00 (150,00; 620,00)
Расчетная СКФ CKD-EPI (>60 мл/мин/1,73 м2); медиана (IQR)	63,00 (54,00; 73,00)	82,50 (73,00; 93,00)	51,00 (49,0; 54,0)	4,0 (4,00; 8,00)	30,50 (17,00; 73,50)
Мин. Т-критерий по результатам ОДМ; медиана (IQR)	-2,60 (-3,0; -1,50)	-1,87 (-3,2; -1,05)	-2,50 (-2,6; -1,5)	-2,35 (-2,90; -1,20)	-1,57 (-2,44; -0,55)

Из 19 пациентов основной группы у 12 при морфологическом исследовании установлена гиперплазия, у 7 — аденома. В группе 1 у всех пациентов установлены аденомы. В группах 2 и 3 у всех диагностирована гиперплазия ОЩЖ.

Сравнительная оценка степени экспрессии CaSR и VDR в зависимости от морфологических изменений в ОЩЖ представлена в таблице 3.

**Table:** Таблица 3. Зависимость экспрессии CaSR и VDR от морфологических изменений ОЩЖTable 3. Dependence of CaSR and VDR expression on PTG morphological changes

Экспрессия рецептора, степень	Аденома n=31 (100%)	Гиперплазия n=104 (100%)	P1	Норма n=5 (100%)	P2	P3
CaSR	0	1 (3,3)	1 (0,9)	>0,1	-	-	-
1	8 (25,8)	14 (13,7)	>0,1	-	-	-
2	17 (54,8)	47 (45,6)	>0,1	-	-	-
3	5 (16,1)	41 (39,8)	≤0,01	5 (100)	≤0,01	≤0,05
VDR	0	13 (41,9)	43 (41,7)	>0,1	-	-	-
1	10 (32,2)	23 (22,3)	>0,1	-	-	-
2	6 (19,3)	24 (23,3)	>0,1	-	-	-
3	2 (6,6)	13 (12,7)	>0,1	5 (100)	≤0,01	≤0,01

Для нормальной ткани ОЩЖ характерна высокая интенсивность экспрессии как CaSR, так и VDR (100%), что можно оценивать как условную «норму». Статистически значимое снижение частоты нормальной экспрессии рецепторов CaSR и VDR встречается при любом морфологическом варианте гиперпаратиреоза и отмечается в 93–60% препаратов. Снижение нормальной экспрессии CaSR при гиперплазии выявляется статистически значимо реже, чем при аденоме (р≤0,01). Медиана интенсивности экспрессии при аденоме составила 2,5 (2–3), при гиперплазии — 3,5 (3–4) (р≤0,01) (рис. 2). Влияние морфологического варианта патологии ОЩЖ на интенсивность экспрессии VDR не обнаружено (p>0,1).

Сравнительная оценка степени экспрессии CaSR и VDR в зависимости от вида гиперпаратиреоза, почечной функции представлена в таблице 4.

**Figure:**
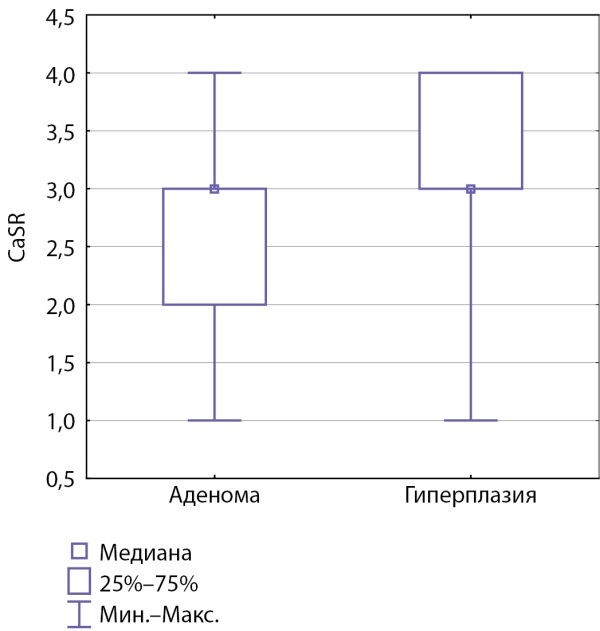
Рисунок 2. Зависимость интенсивности экспрессии CaSR от морфологического варианта патологии ОЩЖ.Figure 2. Dependence of CaSR expression intensity on the morphological variant of PTG pathology.

**Table:** Таблица 4. Степень экспрессии CaSR и VDR в зависимости от вида гиперпаратиреоза, почечной функцииTable 4. The degree of expression of CaSR and VDR depending on the type of hyperparathyroidism, renal function

Экспрессия рецептора, степень	Основная группа Множественное поражение ОЩЖ (n=31; 100%)	Группа сравнения 1.1 Солитарное поражение ОЩЖ рСКФ>60 мл/мин/1,73 м2 (n=18; 100%)	Группа сравнения 1.2 Солитарное поражение ОЩЖ рСКФ<60 мл/мин/1,73 м2 (n=9; 100%)	Группа сравнения 2 ВГПТ на ЗПТ ГД (n=48; 100%)	Группа сравнение 3 ТГПТ на ЗПТ ТП (n=29; 100%)	P1	P2	P3	P4	P5	P6	P7	P8	P9	P10	P11	P12	P13
CaSR	0	1 (3,25)	-	-	1 (2,2)	-	-	>0,1	>0,1	>0,1	-	>0,1	>0,1	>0,1	>0,1	>0,1	>0,1	>0,1	>0,1
1	5 (16,1)	4 (25)	3 (33,3)	4 (8,3)	6 (20,7)	>0,1	>0,1	>0,1	>0,1	>0,1	>0,1	>0,1	>0,1	>0,1	>0,1	>0,1	>0,1	>0,1
2	10 (32,2)	9 (56,25)	4 (44,4)	24 (50)	17 (58,6)	>0,1	>0,1	>0,1	≤0,05	>0,1	>0,1	>0,1	>0,1	>0,1	>0,1	>0,1	>0,1	>0,1
3	15 (48,2)	3 (18,75)	2 (22,3)	19 (39,5)	6 (20,7)	≤0,05	>0,1	>0,1	≤0,05	>0,1	≤0,05	>0,1	>0,1	>0,1	>0,1	≤0,05	≤0,05	>0,1
VDR	0.	12 (38,7)	5 (31,25)	3 (33,3)	22 (45,8)	13 (48,3)	>0,1	>0,1	>0,1	>0,1	>0,1	>0,1	>0,1	>0,1	>0,1	>0,1	>0,1	>0,1	>0,1
1	8 (25,8)	6 (37,5)	3 (33,3)	11 (22,9)	5 (17,2)	>0,1	>0,1	>0,1	>0,1	>0,1	>0,1	>0,1	>0,1	>0,1	>0,1	>0,1	>0,1	>0,1
2.	7 (22,25)	5 (31,25)	2 (22,3)	8 (16,6)	8 (27,5)	>0,1	>0,1	>0,1	>0,1	>0,1	>0,1	>0,1	>0,1	>0,1	>0,1	>0,1	>0,1	>0,1
3	4 (12,95)	-	1 (11,1)	7 (11,7)	2 (7)	>0,1	>0,1	>0,1	>0,1	>0,1	>0,1	>0,1	>0,1	>0,1	>0,1	>0,1	>0,1	>0,1

Частота высокой (3-й степени, нормальной) экспрессии CaSR статистически значимо преобладала при ПГПТ с множественным поражением ОЩЖ (48%) и при ВГПТ на ЗПТ ГД (39%) в сравнении с солитарным поражением при ПГПТ (19–22%) и множественным при ТГПТ на ЗПТ ТП (21%) (p≤0,05).

Выявленные зависимости дают основание определить характерные молекулярно-генетические особенности исследуемых заболеваний, связанные с изменением интенсивности экспрессии CaSR ОЩЖ. Молекулярно-генетический механизм развития солитарных аденом при ПГПТ и множественного поражения при ТГПТ на ЗПТ ТП — снижение частоты сохранения нормальной экспрессии CaSR до 19–21% в отличие от множественного поражения ОЩЖ при ПГПТ и ВГПТ на ЗПТ ГД, при котором установлена более высокая частота сохранения (в 39–48% образцах ОЩЖ) нормальной экспрессии CaSR (p≤0,05).

Для выявления локального и общего характера молекулярно-генетических изменений в экспрессии генов при множественном поражении ОЩЖ оценена вариабельность экспрессии CaSR и VDR в разных ОЩЖ у одного и того же пациента (табл. 5).

**Table:** Таблица 5. Вариабельность экспрессии CaSR и VDR в разных ОЩЖ у одного пациентаTable 5. Variability of CaSR and VDR expression in different PTGs in one patient Примечание. Непрерывные данные представлены в виде медианы и межквартильного диапазона (IQR). Уровень значимости (p) рассчитан по критерию Вилкоксона. Статистически значимые результаты выделены жирным шрифтом. Степени экспрессии перекодированы: 1 — 0-я степень; 2 — 1-я степень; 3 — 2-я степень; 4 — 3-я степень. Сокращения: Мин. — минимальная; Макс. — максимальная.

	Множественное поражение ОЩЖ при ПГПТ nпациентов=19; nпрепаратов=31	ВГПТ на ЗПТ ГД nпациентов=15; nпрепаратов=48	ТГПТ на ЗПТ ТП nпациентов=10; nпрепаратов=31
Мин. степень экспрессии	Макс. степень экспрессии	P	Мин. степень экспрессии	Макс. степень экспрессии	P	Мин. степень экспрессии	Макс. степень экспрессии	P
CaSR	3 (2–3)	4 (4–4)	0,014	3 (2–3)	4 (4–4)	≤0,01	2 (2–3)	4(3–4)	0,023
VDR	2 (1–2)	3 (1–4)	0,025	1 (1–1)	3 (2–4)	≤0,01	1(1–1)	3(3–3)	0,011

Для множественного поражения ОЩЖ при ПГПТ, как и для ВГПТ на ЗПТ ГД и ТГПТ на ЗПТ ТП, характерна неоднородность экспрессии CaSR и VDR в препаратах ОЩЖ от одного и того же больного, что свидетельствует о спонтанном локальном характере молекулярно-генетических нарушений в ОЩЖ в отобранных для анализа наблюдениях (нет случаев генетических наследственных синдромов).

Для оценки вариабельности молекулярно-генетических нарушений в ОЩЖ в зависимости от клинических особенностей гиперпаратиреоза, почечной функции и вида заместительной терапии исследовали вариабельность экспрессии CaSR и VDR в разных ОЩЖ у одного пациента в группах множественного поражения (табл. 6).

**Table:** Таблица 6. Сравнение вариабельности экспрессии CaSR и VDR в разных ОЩЖ одного пациента при различных клинических вариантах ГПТTable 6. Comparison of CaSR and VDR expression variability in different PTGs of one patient with different clinical variants of HPT

	Множественное поражение ОЩЖ при ПГПТ nпациентов=19; nпрепаратов=31	P1	ВГПТ на ЗПТ ГД nпациентов=15; nпрепаратов=48	ТГПТ на ЗПТ ТП nпациентов=10; nпрепаратов=31	P2
Вариабельность экспрессии CaSR	1 (1–2)	0,985	1 (1–1)	1 (0–2)	0,988
Вариабельность экспрессии VDR	1 (0–1)	0,074	2 (1–2)	2 (1–2)	0,050

Вариабельность экспрессии CaSR в ОЩЖ одного и того же больного не зависит от клинических особенностей гиперпаратиреоза, почечной функции и вида заместительной почечной терапии (p>0,95), что свидетельствует о стабильности (или первичности) выявленного молекулярно-генетического механизма при множественном поражении. Вариабельность экспрессии VDR при ПГПТ оказалась ниже, чем у больных, получающих ЗПТ (p<0,07; p<0,05), что свидетельствует об изменчивости выявленного молекулярно-генетического механизма под действием ЗПТ.

Совокупность полученных данных дает основание заключить, что различным молекулярно-генетическим механизмом развития гиперпаратиреоза с преобладанием аденомы (ПГПТ с солитарной аденомой) или гиперплазии (ВГПТ на ЗПТ ГД и ПГПТ с множественным поражением) выявлено различие в частоте сохранения нормальной экспрессии CaSR в ткани ОЩЖ. Этот механизм реализуется на локальном уровне, его вариабельность не изменяется под влиянием ЗПТ.

Общим молекулярно-генетическим механизмом развития гиперпаратиреоза с преобладанием аденомы или гиперплазии установлено снижение частоты сохранения нормальной экспрессии VDR в ОЩЖ (до 7–13%), p<0,01. Этот механизм реализуется на локальном уровне, его вариабельность изменяется под влиянием ЗПТ, достигая статистически значимых различий у больных ТГПТ.

Зависимость характера экспрессии CaSR и VDR от основных лабораторных показателей при ПГПТ с множественным поражением ОЩЖ представлена в таблице 7.

**Table:** Таблица 7. Кросс-таблица на основе ранговых корреляций Спирмена для группы ПГПТ с множественным поражением ОЩЖTable 7. Crosstab based on Spearman's rank correlations for the group of PHPT with multiple PTG lesions Примечание. Наличие сопряженности p<0,05; достоверное отсутствие сопряженности признаков p>0,95.

	CaSR	VDR	Креатинин	Ca, cкоррект. по альбумину	Вит. D	Суточная кальциурия
CaSR	x	0,309	0,476	-0,012	-0,071	-0,321
VDR	0,309	x	0,208	-0,010	0,007	-0,645
Креатинин	0,476	0,208	x	0,156	0,446	-0,336
Ca, cкоррект. по альбумину	-0,012	-0,010	0,156	x	0,314	0,038
Витамин D	-0,071	0,007	0,446	0,314	x	0,133
Суточная кальциурия	-0,321	-0,645	-0,336	0,038	0,133	x

В когорте ПГПТ с множественным поражением ОЩЖ уровень кальция крови не связан с экспрессией CaSR и VDR. Снижение экспрессии CaSR сопряжено с более низким уровнем креатинина крови (сохраненная функция почек).

При множественном поражении ОЩЖ вследствие ВГПТ установлена положительная корреляция между экспрессией CaSR и VDR, отрицательная — между экспрессией VDR и уровнем кальцидиола в крови (табл. 8).

**Table:** Таблица 8. Кросс-таблица на основе ранговых корреляций Спирмена для группы ВГПТ на ЗПТ ТПTable 8. Crosstab based on Spearman's rank correlations for the SHPT group on RRT LT Примечание. Наличие сопряженности p<0,05; достоверное отсутствие сопряженности признаков p>0,95; . ОДМ — остеоденситометрия.

	CaSR	VDR	Витамин D	Кальций ион.	ОДМ
CaSR	x	0,344	-0,187	-0,140	0,203
VDR	0,344	x	-0,408	0,088	0,002
Витамин D	-0,187	-0,408	х	0,219	0,221
Морфология	-0,044	-0,007	-0,273	-0,355	-0,298
Кальций ион.	-0,140	0,088	0,219	x	-0,075
ОДМ	0,203	0,002	0,221	-0,075	x

При сохраненной функции почек уровень кальцидиола не связан с экспрессией VDR на поверхности паратиреоцитов. При ВГПТ на ЗПТ ГД выявляется умеренная отрицательная корреляция между уровнем кальцидиола в крови и экспрессией VDR и положительная — экспрессией CaSR и VDR. Совокупность выявленных корреляций показывает различную роль витамина D в реализации молекулярно-генетических механизмов заболеваний — она не выявляется при сохраненной функции почек и манифестирует у больных на ЗПТ.

## ОБСУЖДЕНИЕ

Исследование оценивает характер экспрессии CaSR, VDR, а также показывает ее зависимость от клинического варианта гиперпаратиреоза, морфологического субстрата и основных лабораторных показателей.

Статистически значимое снижение частоты нормальной экспрессии CaSR и VDR встречается при любом морфологическом варианте гиперпаратиреоза и отмечается в 93–60% случаев. Снижение нормальной экспрессии CaSR при гиперплазии выявляется статистически значимо реже, чем при аденоме (р≤0,01). Медиана интенсивности экспрессии при аденоме составила 2,5 (2–3), при гиперплазии – 3,5 (3–4) (р≤0,01). Полученные в исследовании данные дополняют существующие представления [17–19], показывая не только различия в интенсивности экспрессии CaSR и VDR на поверхности паратиреоцитов при аденоме и гиперплазии, но и частоту снижения экспрессии при различных морфологических вариантах заболевания.

Различие молекулярно-генетических механизмов развития гиперпаратиреоза с преобладанием аденомы (ПГПТ с солитарной аденомой) или гиперплазии (ВГПТ на ЗПТ ГД и ПГПТ с множественным поражением) реализуется в частоте сохранения нормальной экспрессии CaSR в ткани ОЩЖ. Эти механизмы проявляются на локальном уровне, их вариабельность не изменяется под влиянием ЗПТ. Выше показано, что экспрессия CaSR на паратиреоцитах солитарных аденом при ПГПТ снижается до 90% в сравнении с нормальным строением ОЩЖ. Авторы наблюдали снижение CaSR на уровне как белка (рецептора на поверхности паратиреоцитов), так и генов в спорадических аденомах и не обнаружили значимой корреляции между снижением экспрессии рецептора и клинико-лабораторными характеристиками ПГПТ [20]. Результаты исследования показывают, что вариабельность экспрессии CaSR в ОЩЖ одного и того же больного при множественном поражении не зависит от клинических особенностей гиперпаратиреоза, почечной функции и вида заместительной почечной терапии (p>0,95), что свидетельствует о стабильности (или первичности) выявленного молекулярно-генетического механизма.

Общий молекулярно-генетический механизм развития гиперпаратиреоза с преобладанием морфологического субстрата в виде аденомы или гиперплазии — снижение частоты сохранения нормальной экспрессии VDR в ОЩЖ (до 7–13%), p<0,01. Он реализуется на локальном уровне, изменяется под влиянием ЗПТ, достигая статистически значимых различий у больных ТГПТ на ЗПТ ТП. Тaniguchi M. и соавт. установили, что экспрессия CaSR у пациентов с ВГПТ на ЗПТ ГД выше (45,0±2,8), чем при ТГПТ на ЗПТ ТП (29,3±2,3) [21]. В представленном исследовании также получены сведения о снижении частоты нормальной экспрессии CaSR при ТГПТ на ЗПТ ТП. Полученные данные показывают значительную вариабельность снижения экспрессии CaSR в ОЩЖ одного и того же больного. Кроме того, выявлены вариабельность экспрессии VDR в ОЩЖ одного и того же больного с множественным поражением ОЩЖ различного генеза и зависимость этой вариабельности от заместительной почечной терапии: максимальных значений она достигает у больных после трансплантации почки.

Показано значение витамина D в реализации молекулярно-генетических механизмов заболеваний — не выявляется при ПГПТ с сохраненной функцией почек и манифестирует у больных на ЗПТ. Как уже сообщалось, введение кальцитриола или кальцимиметиков приводило к снижению пролиферации клеток ОЩЖ. Это связано с повышением экспрессии как CaSR, так и VDR на фоне введенных препаратов [9][22]. При ПГПТ изменение концентрации кальцидиола не влияет на экспрессию VDR, а уровень кальция не сопряжен с экспрессией CaSR (p>0,95), что позволяет исключить указанные механизмы из патогенеза ПГПТ и не рассматривать медикаментозные методы лечения этого состояния (кальцимиметики и колекальциферол) в качестве патогенетически обоснованных.

Установленная отрицательная корреляция между уровнем кальцидиола в крови и экспрессией VDR у больных на ЗПТ противоречит имеющимся данным о роли хронического дефицита витамина D в развитии гиперплазии ОЩЖ с последующим приобретением автономной секреции ПТГ и трансформацией в аденому при уремическом гиперпаратиреозе [10]. Повышение уровня кальцидиола в крови у включенных в наше исследование больных согласуется со снижением экспрессии VDR. Это противоречие объясняется глубокими локальными молекулярно-генетическими нарушениями, реализующимися в виде согласующейся между собой сниженной экспрессии CaSR и VDR. Ранее было показано, что уже на ранних стадиях развития гиперпаратиреоза недостаточная экспрессия CaSR и VDR делает клетки ОЩЖ неспособными адекватно реагировать на окружающий кальций и/или кальцитриол [12].

Другой причиной выявленного противоречия можно считать развитие автономной секреции ПТГ при низких уровнях экспрессии CaSR. У этих пациентов ограничено использование заместительной терапии дефицита витамина D (повышение уровня кальция крови). В результате уровень кальцидиола в крови больных со сниженной экспрессией CaSR (и, соответственно, VDR), оказывается ниже.

## Ограничения исследования

Исследование имело ряд ограничений: небольшой размер выборки, качественный анализ экспрессии рецепторов, невозможность выполнения анализа экспрессии непосредственно генов рецепторов ввиду отсутствия ресурсов. Главное в проведенной работе заключается в оценке экспрессии основных рецепторов (CaSR, VDR), участвующих в патогенезе всех нозологических вариантов гиперпаратиреоза, а также зависимости экспрессии от варианта гиперпаратиреоза, морфологии и лабораторных показателей. Кроме того, основной акцент сделан на изучение изменений именно множественного поражения ОЩЖ при ПГПТ.

## Направления дальнейших исследований

Дальнейшее исследование экспрессии рецепторов CaSR и VDR на выборке больших размеров с возможным исследованием экспрессии непосредственно генов этих рецепторов поможет лучше понять патогенез множественного поражения ОЩЖ при ПГПТ, что улучшит диагностику и лечение данной формы заболевания.

## ЗАКЛЮЧЕНИЕ

Исследование демонстрирует особенности изменения экспрессии кальций-чувствительного и витамин D рецепторов при ПГПТ с множественным поражением ОЩЖ. Показаны взаимосвязи экспрессии этих рецепторов с клиническим вариантом гиперпаратиреоза, морфологическим субстратом, основными лабораторными показателями и почечной функцией.

## ДОПОЛНИТЕЛЬНАЯ ИНФОРМАЦИЯ

Источники финансирования. Исследование осуществляется в соответствии с планом научно-исследовательской работы (НИР) ИНЦХТ № 063 «Биомедицинские технологии профилактики и лечения органной недостаточности в реконструктивной и восстановительной хирургии», сроки выполнения 2013–2021 гг., а также НИР «Персонифицированный подход к профилактике и коррекции полиорганной дисфункции в лечении хирургических заболеваний», сроки выполнения 2022–2026 гг.

Конфликт интересов. Авторы декларируют отсутствие явных и потенциальных конфликтов интересов, связанных с содержанием настоящей статьи.

Участие авторов. Ильичева Е.А. — разработка концепции и дизайна, анализ и интерпретация данных, обоснование рукописи и проверка критически важного интеллектуального содержания; Шурыгина И.А. — разработка концепции, дизайна иммуногистохимического исследования и интерпретация данных, обоснование рукописи и проверка критически важного интеллектуального содержания; Дремина Н.Н. — проведение иммуногистохимического исследования, анализ и интерпретация данных; Берсенев Г.А. — разработка концепции и дизайна, сбор материала, проведение иммуногистохимического исследования, анализ и интерпретация данных, обоснование рукописи; Григорьев Е.Г.  — разработка концепции и дизайна, анализ и интерпретация данных, обоснование рукописи и проверка критически важного интеллектуального содержания, редактирование и окончательное утверждение рукописи. Все авторы одобрили финальную версию статьи перед публикацией, выразили согласие нести ответственность за все аспекты работы, подразумевающую надлежащее изучение и решение вопросов, связанных с точностью или добросовестностью любой части работы.

Благодарности. Коллектив авторов выражает благодарность Кане Олегу Витославовичу — к.м.н., начальнику ГБУЗ ИОПАБ за помощь в морфологическом исследовании гистологического материала; Махутову Валерию Николаевичу — к.м.н., заведующему торакальным хирургическим отделением ГБУЗ ИОКБ за помощь на всех этапах подготовки рукописи.

1. Immunohistochemistry (IHC): the complete guide: [сайт]. URL: https://www.abcam.com/content/immunohistochemistry-the-complete-guide
